# Effects of vasopressinergic V1 receptor agonists on sublingual microcirculatory blood flow in patients with catecholamine-dependent septic shock

**DOI:** 10.1186/cc9511

**Published:** 2011-03-11

**Authors:** A Morelli, A Donati, C Ertmer, S Rehberg, A Orecchioni, A Di Russo, G Citterio, MR Lombrano, L Botticelli, A Valentini, P Pelaia, P Pietropaoli, M Westphal

**Affiliations:** 1 University of Rome, Italy; 2 Marche Polytechnique University, Ancona, Italy; 3 University Hospital of Münster, Germany

## Introduction

Arginine vasopressin (AVP) and terlipressin (TP) are increasingly used to stabilize mean arterial pressure in the setting of septic shock. Whether these vasopressor agents negatively impact on microcirculatory perfusion is still not fully understood. The objective of the present study was, therefore, to elucidate the effects of AVP and TP on microcirculatory perfusion in patients with catecholamine-dependent septic shock.

## Methods

We enrolled 60 fluid-resuscitated septic shock patients requiring norepinephrine (NE) to maintain mean arterial pressure (MAP) between 65 and 75 mmHg. Patients were randomly allocated to be treated with either continuous TP infusion (1 μg/kg/hour), or AVP (0.04 U/minute), or titrated NE (control; each *n *= 20). In both the TP and AVP groups, NE was titrated to achieve a MAP between 65 and 75 mmHg. Data from right heart catheterization and sidestream dark-field imaging were obtained at baseline and after 6 hours.

## Results

No significant differences were found between groups in terms of MAP, cardiac index, mixed-venous oxygen saturation, arterial lactate, and microvascular flow index of the small vessels (2.1 (1.8; 2.4) vs. 3.0 (2.6; 3.0) for TP, 1.9 (1.7; 2.3) vs. 2.7 (2.0; 3.0) for AVP, 2.3 (2.1; 2.6) vs. 3.0 (2.9; 3.0) for NE). Conversely, AVP and TP significantly reduced NE requirements over time (0.57 (0.29; 1.04) vs. 0.16 (0.03; 0.37) μg/kg/minute for TP and 0.40 (0.20; 1.05) vs. 0.23 (0.03; 0.77) μg/kg/minute for AVP; all *P *< 0.05). However, no differences were found between TP and AVP after 6 hours.

## Conclusions

The results of the present study suggest that vaso-pressinergic V1 agonists allow a reduction in catecholamine requirements without negative impact on microvasular perfusion as compared with sole NE therapy.

